# Supplementary figures and images for: Geographically weighted regression analysis of anemia and its associated factors among reproductive age women in Ethiopia using 2016 demographic and health survey
Source: PLoS One. 2022 Sep 22;17(9):e0274995. doi: 10.1371/journal.pone.0274995 (PMC9498958; doi:10.1371/journal.pone.0274995)

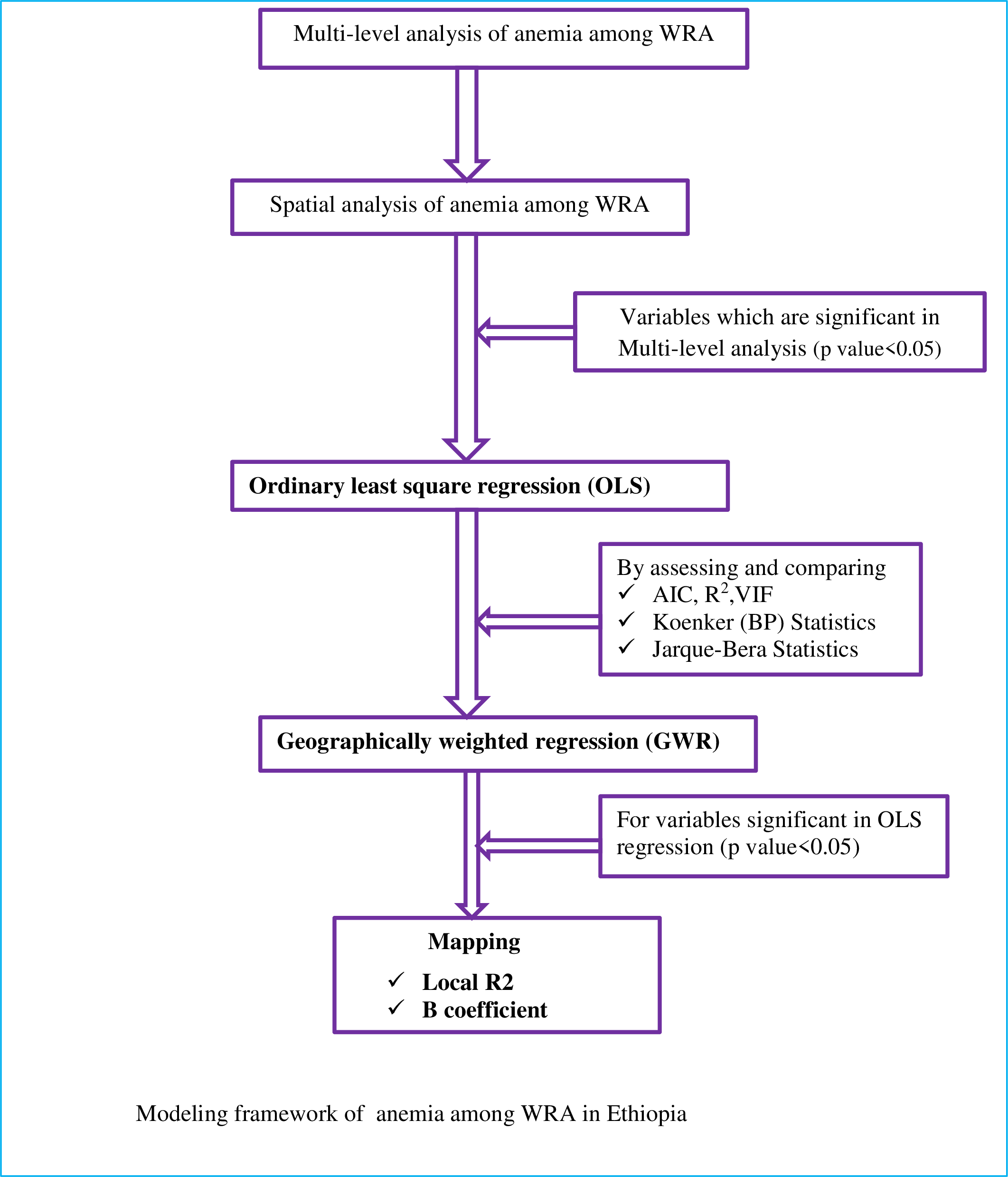

Supplement: S1 Fig — (TIF) [file pone.0274995.s001.tif]
