# Supplementary material for: Geographically weighted regression analysis of anemia and its associated factors among reproductive age women in Ethiopia using 2016 demographic and health survey
Source: PLoS One. 2022 Sep 22;17(9):e0274995. doi: 10.1371/journal.pone.0274995 (PMC9498958; doi:10.1371/journal.pone.0274995)
Supplement: S1 File — (DOCX) [file pone.0274995.s002.docx]

**Acronyms and abbreviations**

CI ---- Confidence interval

CSA -----Central Statistical Agency

DHS ----- Demographic and health survey

EAs----- Enumeration areas

EDHS ----- Ethiopian demographic and health survey

FMoH ----- Federal Ministry of Health

GWR----- Geographical weighted regression

Hb ----- Hemoglobin

HSTP ----- Health Sector Transformation Plans

ICC -----Intracluster correlation coefficient

IDA ----- Iron deficiency anemia

LLR------ log likely hood ratio test

MOR -----Median odds ratio,

OLS-----Ordinary least square

OR------Odds ratio

PCV----- Proportional change invariance

USAID ----- United States of America aid

WHO ------ World health organization

WRA ------Women reproductive age
