# Supplementary material for: Geographically weighted regression analysis of anemia and its associated factors among reproductive age women in Ethiopia using 2016 demographic and health survey
Source: PLoS One. 2022 Sep 22;17(9):e0274995. doi: 10.1371/journal.pone.0274995 (PMC9498958; doi:10.1371/journal.pone.0274995)
Supplement: Attachment — Submitted filename: PLOS ONE Review2.docx [file pone.0274995.s003.docx]

PLOS ONE Review: 10 March 2021

**Geographically weighted regression analysis of anemia and its associated factors among reproductive age women in Ethiopia using the 2016 Demographic and Health Survey.**

**Abstract**

The defininition of anaemia is not clearly defined. I suggest the authors to specificy the actual threshold in g/dL as is defined by the WHO or the Ethiopian Ministry of Health.

The conclusion should clearly speak more on the policy imperatives amidst the disparities highlighted.

**Introduction**

The introduction should re-written to capture the global, SSA and national dynamics of anaemia in Ethiopia. This should be followed by the epidemiological significance of anaemia in Epthiopia.

The clinical defininition of anaemia is not concise. I suggest the authors to specificy the actual threshold in g/dL as is defined by the WHO or the Ethiopian Ministry of Health. Less than normal is not quite clear.

The full definition of WRA is not outlined when it is first mentioned.

I suggest and English editor.

**Materials and methods**

The authors indicate that a fixed effects were used to estimate the association between the likelihood of anaemia. They futher indicate that the random effects were used to estimate the median odds ratio.

We expect the authors to detail the regression model and outline how the fixed and random effects are to be conceptualized.

Some terms used in the defining the model parameters are not defined e,g ICC, VA.

A supplemntary file of the modelling framework will be useful.

I am not sure if ordinary least square analysis as is expreessed is appropriate for the methods section.

**Results**

This section should be rewritten and the model output discussed as per the methodology previously employed.

Whereas there are many factors associated with anaemia from the multi-level analysis in Table 3. The authors have only discussed the age of the women and how it relates to ananemia. Futhermore the wording and odds-ration interpretation is not clear and need to be looked at possibly with the help of a bio-statistician.

The seems to be too many figures in the manuscripts (Figure 1 – Figure 13). I suggest that some figures could be added as a supplementary.

**Discussion**

We need to know more on how the study findings relate to other settings in SSA as compared to Ethiopia.
