# Supplementary material for: Geographically weighted regression analysis of anemia and its associated factors among reproductive age women in Ethiopia using 2016 demographic and health survey
Source: PLoS One. 2022 Sep 22;17(9):e0274995. doi: 10.1371/journal.pone.0274995 (PMC9498958; doi:10.1371/journal.pone.0274995)
Supplement: Attachment — Submitted filename: Response to editor.docx [file pone.0274995.s004.docx]

**Date: August 9/ 2021**

**Response to editor’s comment**

Manuscript title: **Geographically weighted regression analysis of anemia and its associated factors among reproductive age women in Ethiopia using the 2016 Demographic and Health Survey.**

**Manuscript number:** **PONE-D-20-35910**

Dear editor, thank you for the most important issues you raised for the betterment of our manuscript. Below are the response for the comments and concerns you raised. In addition, we have incorporated the comments and concerns in the revised manuscript.

**Response to editor’s comments**

**Response to Editors**

Q1. We note that Figure 4,7,8,9,10,11,12 & 13 in your submission contain map images which may be copyrighted. All PLOS content is published under the Creative Commons Attribution License (CC BY 4.0), which means that the manuscript, images, and Supporting Information files will be freely available online, and any third party is permitted to access, download, copy, distribute, and use these materials in any way, even commercially, with proper attribution.

**Authors’ response:** Dear editor thank you for your important comments.

- These figures are not copyedited from other sources rather we have done using Arc-GIS version 10.7 and SaTScan version 9.6 statistical software by accessing the shapefiles of Ethiopia from <https://open.africa/dataset/africa-shapefiles>. Besides, the geographic coordinate data (latitude and longitude data) were accessed from the Measure DHS program by explaining the objectives of the study. Therefore, we kindly inform you the figures/maps reported in this manuscript are our works which mean not copyedited or not directly taken from other sources.
- The shape file data have been taken from the freely available page, open Africa shape file center. with the web address [*https://open.africa/dataset/africa-shapefiles*](https://open.africa/dataset/africa-shapefiles)
- We have used ArcGIS version 10.7 software, ArcMap10.7 package which is a freely available software with the web address <https://desktop.arcgis.com/en/arcmap/latest/get-started/installation-guide/introduction.htm>
- The coordinate data has been taken from measures DHS after writing a concept note to DHS program and getting permission to use it. <https://dhsprogram.com/Countries/Country-Main.cfm>

Then by using the above software’s and data sets we have plotted the maps by ourselves. For all the software’s, data sets that we used were taken consents from respected address and well expresses in the method sections of the manuscripts.

Q2. About text Overlap

You said that we note that the current submission still contains some textual overlap with other previously published works.

**Authors’ response:** Dear editor thank you for your important comments. We have cheeked the text overlap in all sections of the manuscript and we have made a lot of correction on it. We have given a serious concern and amend the text overlaps in all the manuscript sections.
