# Supplementary material for: Geographically weighted regression analysis of anemia and its associated factors among reproductive age women in Ethiopia using 2016 demographic and health survey
Source: PLoS One. 2022 Sep 22;17(9):e0274995. doi: 10.1371/journal.pone.0274995 (PMC9498958; doi:10.1371/journal.pone.0274995)
Supplement: Attachment — Submitted filename: Response to Reviewers.docx [file pone.0274995.s005.docx]

**Date: January 05/ 2022**

**Response to editor’s comment**

Manuscript title: **Geographically weighted regression analysis of anemia and its associated factors among reproductive age women in Ethiopia using the 2016 Demographic and Health Survey.**

**Manuscript number:** **PONE-D-20-35910**

Dear editor, thank you for the most important issues you raised for the betterment of our manuscript. Below are the response for the comments and concerns you raised. In addition, we have incorporated the comments and concerns in the revised manuscript.

**Response to editor’s comments**

**Introduction section**

- There is an error in the anemia units. The definition should be 120 g/L not g/dL.

**Authors’ response:** Dear editor thank you for your important comments. We revised it as hemoglobin level **<11g/dl** for lactating or pregnant mothers and hemoglobin **level <12 g/dl and** for none pregnant or non-lactating women which means 120 g/L.

- Please run a full spelling check on the manuscript. E.g. see typos in the abstract: “identifay” -> identify and main text “pridictors” -> predictors.

**Authors’ response:** Dear editor thank you for your concern. We have cheeked all the manuscript about typographic errors and we gave a serious concern and amend in all the manuscript sections.

- Line 50 suggest rewording “Reproductive age women are women who are found in the age from 15 up to 49 full-term years”to:Reproductive age is commonly defined among women as ages 15 to 49 years.
- Line 57: suggest rewording: “It is one of the most serious dangers to children's health and a factor in maternal mortality, because it increases the risk of adverse pregnancy outcomes, child mortality, impaired neurocognitive abilities, and physical development of children, and reduces work capacity despite being straightforward to prevent and treat.

**Authors’ response:** Dear editor thank you for your comments. We have revised and correct all the comments you raised in line 50 and 57 based on your suggestions. Line 49…and line 57.

- Line 69: “the pooled prevalence of anemia in pregnant women was 31.66% in Ethiopia” Do you have the year for this metric?

**Authors’ response:** Dear editor thank you for your comments. Corrected as “The pooled prevalence of anemia among pregnant women from 2003 to 2016 was 31.66% in Ethiopia”. Line 68-69.

- Line 70: replace “lactating women” with ‘current lactation’
- Line 73: small ‘a’ for “Anemia’ – please correct this throughout the text.
- Line 83: “supplying”? (not supplaying)

**Authors’ response:** Dear editor thank you for your suggestion. Line 70, 73 and 83 corrected accordingly in the revised manuscript.

**Methods**

- Line 104: “de juries” please define.

**Authors’ response:** Dear editor thank you for your suggestion. Women who were usually live in the surveyed households are known as de juries. defined in line 104.

- Line 118 “khat” please define.

**Authors’ response:** Dear editor thank. Khat chewing is chewing of **stimulant leaves**, which means it speeds up the messages going between the brain and the body. Defined in revised manuscript line 118 as “stimulant plant”.

- Line 126 “we recode them based on the appropriate measure of central tendency” This is unclear – what does it mean? Centralise / normalise based on mean and SD?

**Authors’ response:** Dear editor thank you for your comments. We revised as “The normal distribution of aggregated community factors was assessed by histogram and Shapiro Wilks test but, they didn’t fulfill the normality assumption then we recode them based on the median value”. Line 125-128.

- Line 149: “used for parameter estimation” please change to ‘included in the model’.
- Line 150: “among reproductive age between clusters” Please change to “among reproductive age women between clusters.

**Authors’ response:** Dear editor thank you for your comments. Line 149-150 are revised according to your suggestions.

- Line 151. This does not quite make sense as the metrics you are mentioning are not the same as random effects. Please reword “It was estimated by…” to “We estimated the…”

**Authors’ response:** Dear editor thank you for your comments. Revised as “We used cluster number variable (v001) for random effect estimates. We estimated the intraclass correlation coefficient (ICC)………,.” Line 153-155.

- Line 156: please change “that would have if” to ‘associated with’.
- Line 163: I did not follow this sentence, I think it could be removed: “In this study, since the ICC was greater than 5% and the MOR have significant difference, doing the multilevel analysis was necessary”  as the methods are already clear.

**Authors’ response:** Dear editor thank you for your comments. Revised accordingly line 156 rephrased and line 163 removed.

- Line 200. Sentence seems incomplete: “Unlike OLS that fits a single linear regression equation to all of the data in the study area, GWR 200 creates an equation for each.” …. Each?

**Authors’ response:** Dear editor thank you. Completed as “for each coefficient”.

**Result**

- Table 1: please indicate what the numbers in brackets are.

**Authors’ response:** Dear editor thank you. Revised as percentage (%)

- Line 233: this sentence needs rewording, grammar is incorrect and meaning unclear: “The MOR value in the null model also showed that, between higher and lower odd clusters anemic among reproductive-age women were different by 2.28 times.”

**Authors’ response:** Dear editor thank you. Paraphrased as “The MOR value in the null model also showed that, anemic among reproductive-age women were different by 2.28 times between higher and lower prevalence clusters”.

- Fig 3: Pia chart – do you mean Pie chart?
- Line 236 replace “expressed” with ‘explained’.
- Line 286: “from these 50 of these”  please rephrase to ’50 of these’
- Line 297: “kiringing” correct to “kriging” (I think?)
- Line 303 missing figure number.

**Authors’ response:** Dear editor thank you for your comments. All the above comments revised and paraphrased accordingly.

**Discussion**

- Line 358: suggest deleting this phrase which does not make sense to me “during their reproductive cycle end” since the paper covers anaemia among pregnant women.

**Authors’ response:** Dear editor thank you for your comments. Deleted the phrase.

- Line 371: this sentence is unclear please change: “Moreover, the number of cluster differences between EDHS has its effect[10, 28].”

**Authors’ response:** Dear editor thank you for your comments. This is to explain the sample size difference might have effect on prevalence. Revised as “Moreover, the number women included in each EDHS might have its own effect”

- Line 394 please replace “women who were rich have less likely” with ‘women who were rich are less likely’- **Corrected accordingly**.
- Line 396-403 – many grammatical errors here, please check and correct.

**Authors’ response:** Dear editor thank you. Corrected as “This could be due to that, when a woman has improved wealth status, she enables to purchase healthy nutrition, and can utilize health services”

- Line 419 please correct the grammar in this sentence: “However, being women having more than 1 child within five years had a positive relationship with anemia among WRA in different regions of Ethiopia.” To “However, women who had more than 1 child within five years were more likely to have anemia in multiple regions of Ethiopia.”
- Line 434: please change “Therefore, the Ethiopia FMoH is important to pay special” to “Therefore, it is important that the Ethiopia FMoH pay special”
- Line 437-438 please correct “should be a strength” to “should be strengthened”.

**Authors’ response:** Dear editor thank you for your paraphrasing. Line 419,434, and 437 are corrected according to your suggestion.

- Fig 1 define Hgh

**Authors’ response:** Dear editor thank you for concern. We revised the figure and define all the terms. HGB- Hemoglobin, WRA- Women in Reproductive Age, and De jure - Women who usually live in the surveyed households.

- Figures 2, 3 correct title: trend of anaemia ‘in’ reproductive women.
- Figure 4 spell check title.

**Authors’ response:** Dear editor thank you for your comment. Spell cheeked and corrected accordingly.

- Figure 7 – no cold spots are visible.

**Authors’ response:** Dear editor thank you for your comment. There are significant cold spot areas of 90% confidence interval, but there were no significant cold spot area of 95% and 99% confidence interval.

- Figure 8 and 9 seem to be duplicates?

**Authors’ response:** Dear editor thank you for your comment. Yes you are correct, the image 8 was error. We revised the and plot the new image 8
